# Surgical planning in virtual reality: a systematic review

**DOI:** 10.1117/1.JMI.11.6.062603

**Published:** 2024-04-25

**Authors:** Moritz Queisner, Karl Eisenträger

**Affiliations:** a Charité – Universitätsmedizin Berlin, Department of Surgery, Experimental Surgery, Berlin, Germany; b Humboldt Universität zu Berlin, Cluster of Excellence Matters of Activity, Berlin, Germany

**Keywords:** surgery, virtual reality, planning, systematic review

## Abstract

**Purpose:**

Virtual reality (VR) technology has emerged as a promising tool for physicians, offering the ability to assess anatomical data in 3D with visuospatial interaction qualities. The last decade has witnessed a remarkable increase in the number of studies focusing on the application of VR to assess patient-specific image data. This systematic review aims to provide an up-to-date overview of the latest research on VR in the field of surgical planning.

**Approach:**

A comprehensive literature search was conducted based on the preferred reporting items for systematic reviews and meta-analyses covering the period from April 1, 2021 to May 10, 2023. It includes research articles reporting on preoperative surgical planning using patient-specific medical images in virtual reality using head-mounted displays. The review summarizes the current state of research in this field, identifying key findings, technologies, study designs, methods, and potential directions for future research.

**Results:**

The selected studies show a positive impact on surgical decision-making and anatomy understanding compared to other visualization modalities. A substantial number of studies are reporting anecdotal evidence and case-specific outcomes. Notably, surgical planning using VR led to more frequent changes in surgical plans compared to planning with other visualization methods when surgeons reassessed their initial plans. VR demonstrated benefits in reducing planning time and improving spatial localization of pathologies.

**Conclusions:**

Results show that the application of VR for surgical planning is still in an experimental stage but is gradually advancing toward clinical use. The diverse study designs, methodologies, and varying reporting hinder a comprehensive analysis. Some findings lack statistical evidence and rely on subjective assumptions. To strengthen evaluation, future research should focus on refining study designs, improving technical reporting, defining visual and technical proficiency requirements, and enhancing VR software usability and design. Addressing these areas could pave the way for an effective implementation of VR in clinical settings.

## 1 Introduction

**Fig. 1:**
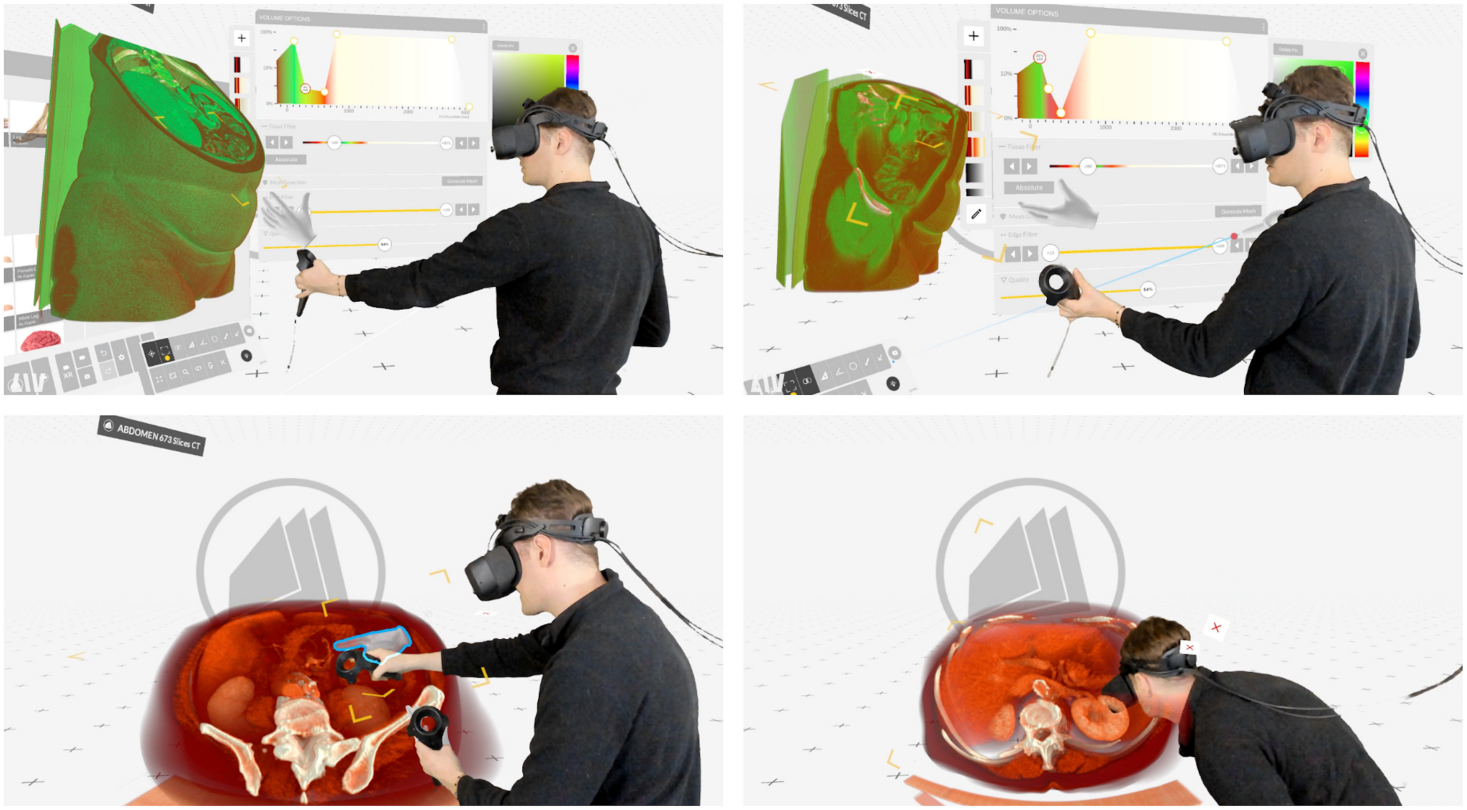
VR Software Medical Holodeck (Medical Imaging XR, Zürich, Switzerland)^4^ used to render a CT of an abdomen.

The concept of virtual reality (VR) is based on a type of computer-generated imagery that is particularly useful to convey space and spatial relations. Conventional screens present images in a two-dimensional (2D) format, limiting depth perception. In contrast, VR head-mounted displays (HMDs) or specific 3D displays can visualize images in three dimensions (3D), which allows physicians to experience anatomical data with visual depth cues, from every angle and on any scale. Stereoscopic visualization makes VR an ideal medium for the visualization of volumes. Although 3D images can be displayed on a standard 2D monitor as well, they lack the ability to display depth. In addition to its capability for 3D visualization, VR enables interaction with images to be embodied: VR sensing technology synchronizes translational movements (forward and backward, up and down, left and right) and rotational movement (tilting sideways, forward and backward, and left and right) of the user’s body in the virtual environment. Due to this dynamic adjustment of medical image data to movement and changes in position, VR HMDs offer an additional layer of sensory input compared to other 3D image visualization modalities. This additional input is constituted by vestibular and proprioceptive feedback information, which mimics natural interaction with actual volumes. The ability of VR images to convey spatial information in connection to its adaptability to the user’s sensorimotor system is assumed to improve spatial understanding and reasoning based on the concept of embodied cognition.^1^^–^^3^ This allows physicians to experience anatomical data from every angle and on any scale (Fig. 1).

Most medical imaging modalities generate volumetric data, but this data is usually displayed using reformatted planes (axial, sagittal, coronal planes) on conventional monitors. However, surgical decisions rely on the understanding of volumes and their spatial relations to each other. They depend on mental models of three-dimensional structures derived from two-dimensional images. Imaging technologies, such as computed tomography (CT) or magnetic resonance imaging (MRI), gather volumetric data that is usually used to visualize spatial structures of a body as tomographic images. In surgical planning, physicians need to rely on their visual expertise and imagination to bridge the gap between tomographic slices (2D) and anatomy (3D) they handle during surgery. Accordingly, a surgical decision in the planning process is based on a convergence of graphical aptitude and anatomical expertise that surgeons traditionally perform cognitively. This requires profound and enduring training and carries the risk that 2D images may not be adequately translated into mental representations and in consequence onto the patient’s body.

**Fig. 2:**
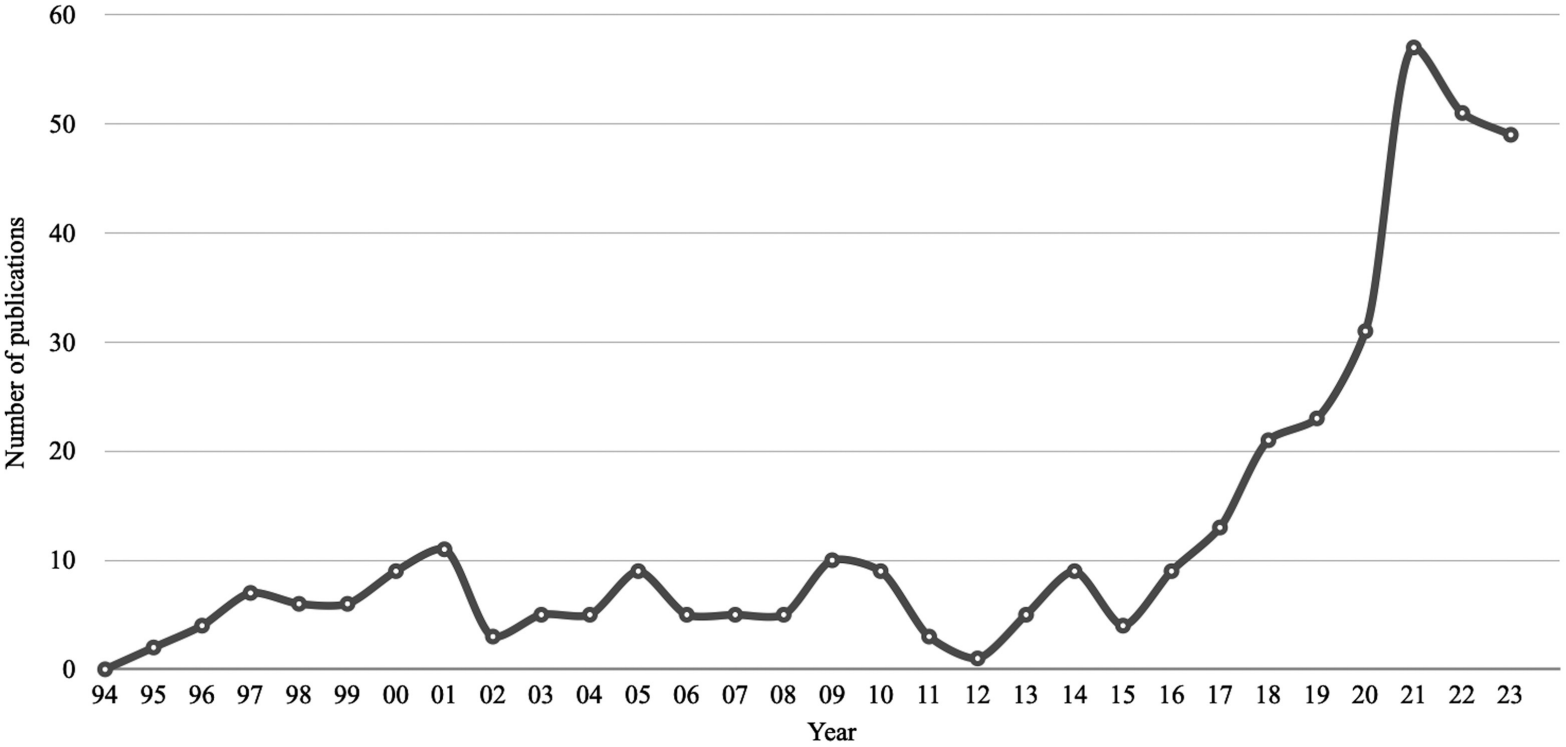
Number of publications per year for PubMed search query “(virtual reality[Title/Abstract]) AND (surgery[Title/Abstract])) AND (planning[Title/Abstract]).” The values for 2023 contain data until May 2023, which is why the graph shows a decreased value for that year.

It is by no means a new concept to apply VR in medical imaging to facilitate the translation between image and patient. However, the improved capabilities of imaging, sensor, and display technology as well as the availability of VR-specific surgical planning software make VR more suitable for clinical practice. This corresponds to the rapidly growing number of VR-related research papers on surgical planning in the last 5 years (Fig. 2). Several systematic reviews have analyzed surgical planning in virtual reality with regard to specific surgical disciplines or specific procedures and cases.^5^^–^^10^ This review seeks to provide a systematic overview of VR-based planning for the whole domain of surgery. One recent review has addressed this general perspective before, analyzing data until March 2021.^11^ By taking into account research papers since April 1, 2021, we provide updated data on the rapidly changing landscape of VR technology for preoperative surgical planning and compare the results with the previous review.

## 2 Methods

This review adhered to the preferred reporting items for systematic reviews and meta-analyses (PRISMA) statement.^12^

### 2.1 Search Strategy

**Fig. 3:**
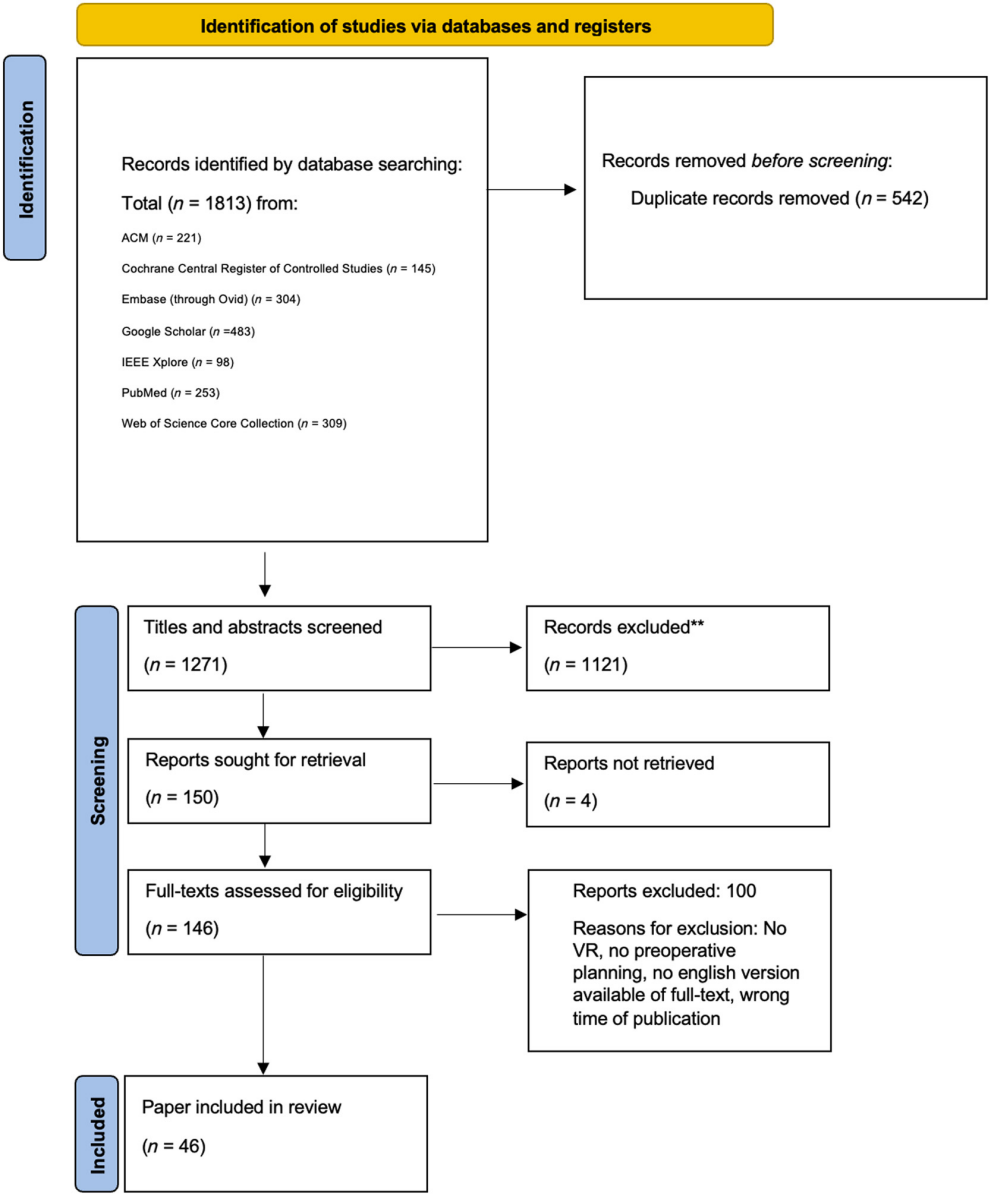
Search strategy based on the PRISMA flowchart.

A literature search was performed to identify relevant research articles written in English and listed in the following databases: the Association for Computing Machinery Digital Library, CENTRAL, Embase, Google Scholar, IEEE Xplore, PubMed, and Web of Science Core Collection (Fig. 3). Results were limited to the timeframe between April 1, 2021, and May 10, 2023. All searches were conducted on May 10, 2023. The search strategy employed specific keywords: [(“VR or virtual reality” or “virtual-reality”) and (“surgery” or “operation” or “surgical”) and (“planning” or “pre-operative” or “preoperative” or “pre-op” or “presurgical” or “pre-surgical” or “preplanning” or “pre-planning”)]. A detailed overview of the search methodology and queries for each database is summarized in Table S1 in the Supplementary Material.

### 2.2 Eligibility Criteria

Eligible studies included the use of immersive VR with an HMD for preoperative surgical planning with patient-specific medical images. The condition for patient-specific data was fulfilled if the studies employed patient-specific data extracted from the individual’s physical body to generate VR volumes. Studies using generic patient data were not considered. Reviews, editorials, opinion-based articles, abstracts without full-text articles, videos without full-text articles, and tutorials were also excluded from the analysis.

### 2.3 Screening and Study Selection

Two independent reviewers (the authors) conducted a screening of titles and abstracts in the bibliography software Zotero.^13^ The initial step was to manually exclude any duplicate entries. Subsequently, the titles and abstracts were independently assessed, followed by an independent evaluation of the full-text articles. Cases of different judgments were resolved through discussion.

### 2.4 Data Extraction and Analysis

We used a custom data extraction template. The following data were extracted from the selected research articles: study design, surgical discipline, procedure or indication, VR software utilized, HMD employed, medical imaging technique that provided data for the input of the VR model, visualization modality compared with VR, order of presentation of the different visualization modalities, number and specialization of participants, number of cases studied, outcome variables measured, and whether the research findings favored VR over the compared visualization modalities. All extracted data were noted in a sheet in Table S2 in the Supplementary Material.

## 3 Results

### 3.1 Search Results

The initial search yielded a total of 1813 studies. After removing duplicates, we reviewed the remaining 1271 studies by scanning their titles and abstracts to assess if they met the criteria, further reducing the number of studies to 150. Those 150 articles were reviewed in full-text, 46 of them fulfilled the eligibility criteria.

### 3.2 Study Characteristics: Study Design, Surgical Disciplines, Participants, and Cases

The 46 articles featured 52 studies with the following study designs: experiments (9 of 52), usability studies (9 of 52), retrospective reviews (7 of 52), case series (6 of 52), single-case reports (6 of 52), prospective observational case series (5 of 52), pilot studies (3 of 52), software elaborations (3 of 52), randomized controlled trials (2 of 52), a proof of concept study (1 of 52), and a feasibility study (1 of 52).

**Fig. 4:**
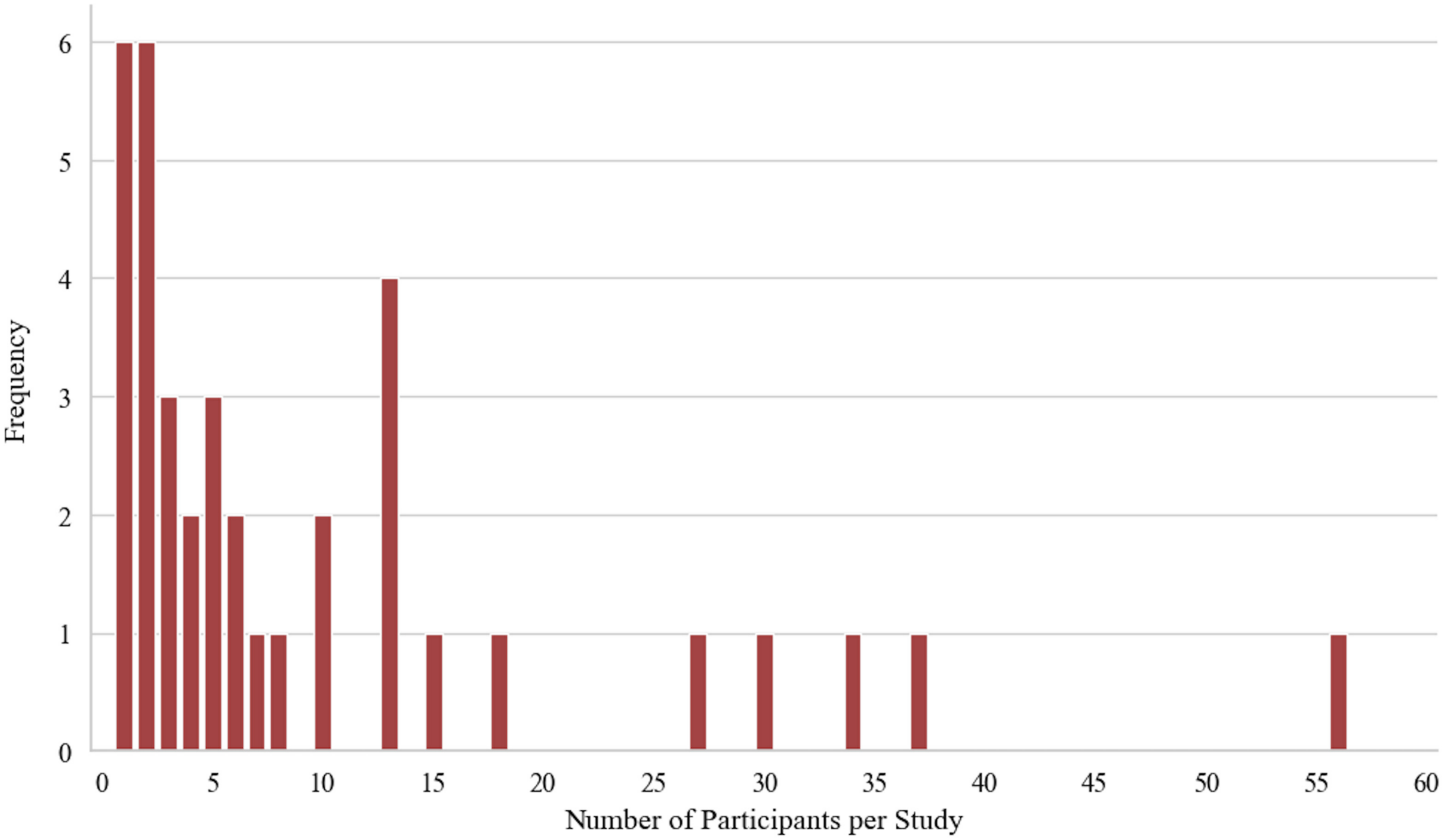
Number of participants per study.

The articles covered the following surgical disciplines (Fig. 4): cardiothoracic surgery (20 of 52), general surgery (11 of 52), neurosurgery (10 of 52), oral and maxillofacial surgery (5 of 52), orthopedic surgery (2 of 52), otorhinolaryngologic surgery (2 of 52), plastic surgery (1 of 52), and urology (1 of 52).

**Fig. 5:**
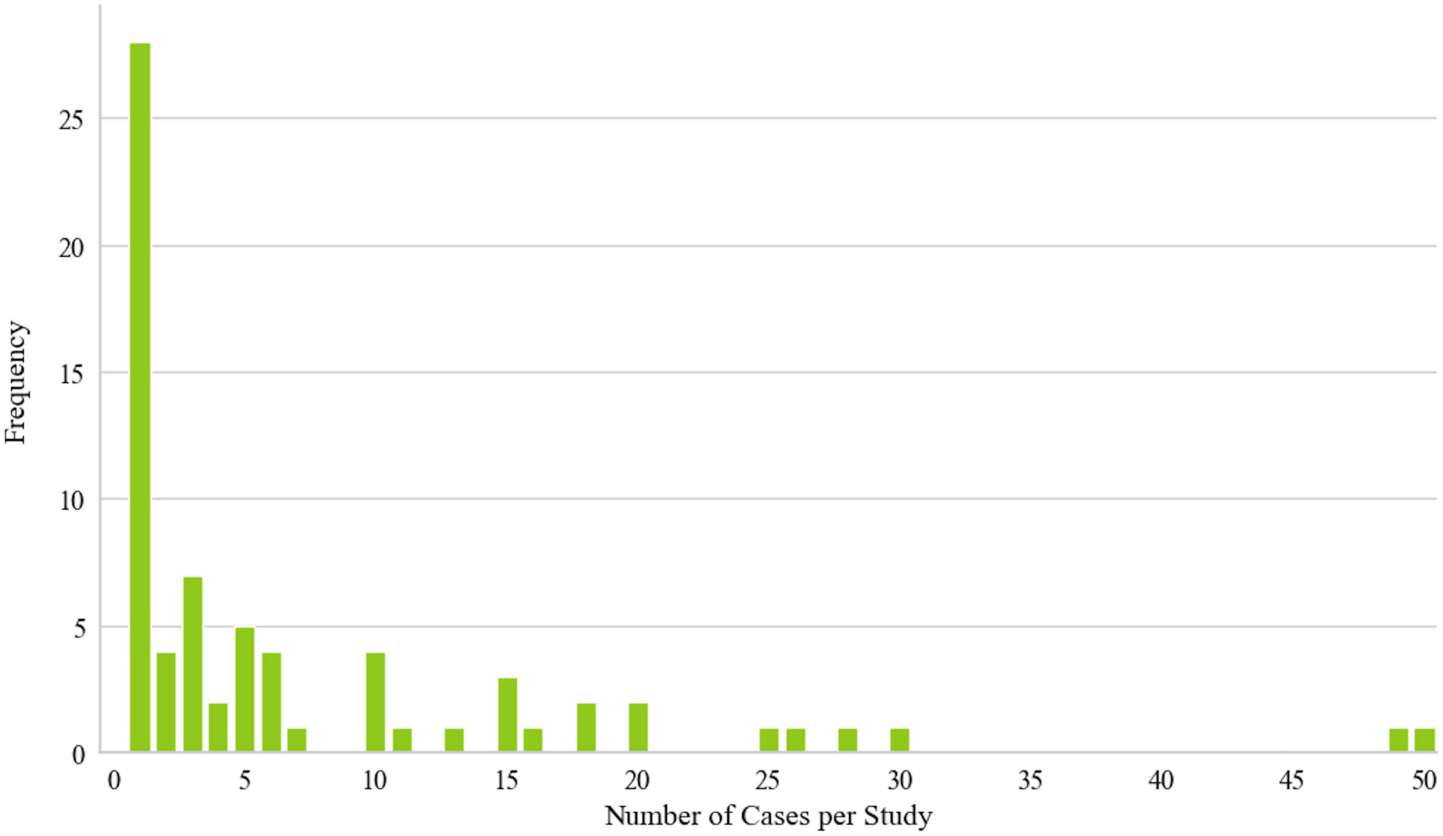
Number of cases per study.

The average number of participants per study was 9.89 (±12.13) with a median of 5. Figure 4 indicates the frequencies of participants per study. In 15 instances, the number of participants was not stated. The average number of cases per study was 10.13 (±11.76) with a median of 5. Excluding the single-case studies, the average rises to 13.09 (±12.13) with a median of 10. Figure 5 shows the frequencies of cases for all studies. Seven studies did not provide the number of cases.

### 3.3 Methods of Comparing Different Visualization Modalities

Comparisons between VR and other visualization modalities were assessed in different ways. One way was to conduct planning based on one visualization modality and then update it after seeing another visualization modality. Other articles assessed planning in VR and with other modalities separately. In 20 cases, no comparisons were made at all. In 23 cases, VR was compared to 2D data displayed on a regular monitor, in seven cases to 3D data displayed on a monitor, and in four cases 2D and 3D data displayed on a monitor. In six cases, VR was compared to 3D printed models. In one instance, VR was compared to the use of a monitor displaying 2D data supplemented with 3D printed models.

### 3.4 Image Generation, Segmentation, and Rendering

The following input data and imaging modalities were used in the studies for the generation of VR images (Fig. 5): CT (24 of 52), MRI (11 of 52), CT and MRI (11 of 52), dynamic 3D echocardiography (3 of 52), CT and facial scan (1 of 52), and CT and digital subtraction angiography (1 of 52). One study did not specify the medical imaging technique used to generate the data for the VR model. Eight datasets used for VR were not segmented. In one study, data were segmented in VR. Eight out of 43 segmented datasets were manually segmented, three were automatically segmented, and five were semiautomatically segmented. In the remaining 27 studies, it was not clear if the segmentation was done manually, semiautomatically, or automatically. The deployed rendering techniques were rarely stated explicitly. Therefore, we often needed to identify which technique was used from the images or videos provided by the studies. 19 studies used only meshes, 15 used volumetric rendering, and 18 used a mixture of volumetric rendering with meshes inserted.

### 3.5 Virtual Reality Hardware and Software

**Fig. 6:**
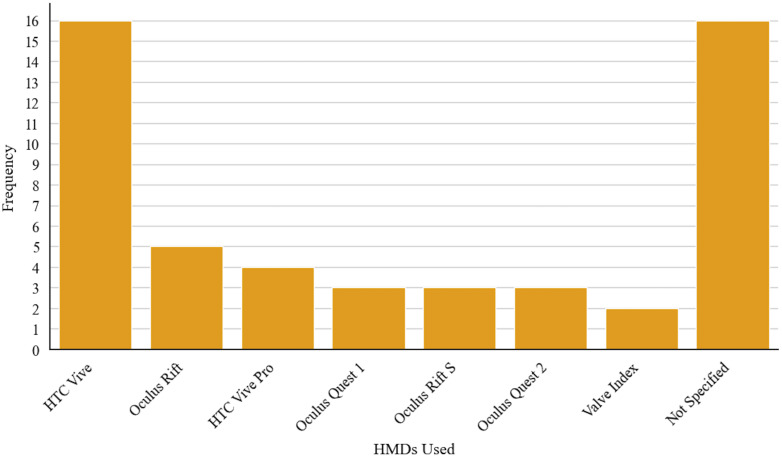
Number of the type of HMDs used in the selected studies.

3D-VR models were displayed with the following commercially available HMDs: HTC Vive (16 of 52), Oculus Rift (5 of 52), HTC Vive Pro (4 of 52), Oculus Quest 1 (3 of 52), Oculus Quest 2 (3 of 52), Oculus Rift S (3 of 52), and Valve Index (2 of 52) (Fig. 6). In 16 instances, the model of the HMD was not specified. In all cases, the HMDs were tethered to a computer; no study stated explicitly that they used an HMD in standalone mode. In 19 of the 52 studies, specifications of the tethered computer were indicated. In 12 of these, the random-access memory (RAM) was specified. In 18 studies, the graphics processing unit (GPU) was named by model series, but in 11 cases, the video random-access memory (VRAM) of the GPU was not provided. In 17 studies, the processor series was mentioned.

The studies used the following VR software: custom-made software (18 of 52) Surgical Theater^14^ (9 of 52), MedicalVR^15^ (7 of 52), Specto VR^16^ (3 of 52), DIVA^17^ (2 of 52), Enduro^18^ (2 of 52), SlicerVR^19^ (1 of 52), Adesante SurgeryVision^20^ (1/52), adapted version of IMHOTEP^21^^,^^22^ (1/52), BananaVision^23^ (1/52), CorFix^24^ (1 of 52), Elucis (1 of 52), Marion K181 PCNL simulator^25^ (1 of 52), nonmedical beta test version of software AW Virtual Reality prototype^26^ (1 of 52), 3D Systems^27^ (1 of 52), and VisuaMed^28^ (1 of 52). One study did not indicate the software that was used.

### 3.6 Surgical Planning Changes and Outcomes

We distinguished six groups of studies, based on combinations of study type and outcome data: retrospective reviews on preoperative planning changes, retrospective reviews on surgical outcomes, prospective studies on preoperative planning changes, prospective studies on surgical outcomes, case studies and case series, and comparative studies. The following sections summarize the outcomes of the identified groups.

#### 3.6.1 Retrospective reviews on preoperative planning changes

Milano et al.^29^ conducted a study involving 10 consecutive patients with complex double outlet right ventricle and complex interventricular communications, who underwent biventricular repair; an arterial switch operation was part of the repair in three of those. In their study, they asked two experienced pediatric cardiac surgeons, which were unfamiliar with the cases, to review every case and had them decide on a surgical plan. The surgeons reached an agreement of 75% with actual surgical plans after reviewing CT/MRI scans on screens. When using a 3D PDF file, a specific file type that can contain geometric information and can be rotated and zoomed, they only identified the actual plan in 70% of cases. When viewing 3D-printed models, the accordance increased to 85%. By assessing the cases in VR, the accordance improved further to 95%.

A retrospective study by van de Woestijne et al.^30^ compared preoperative plans newly devised by two surgeons with the help of CT and VR with the actual operative plans. The surgeons were blind to the original plans. In 57% (4 of 7) cases, they developed a different preoperative plan or made new observations. Thumerel et al.^26^ conducted a retrospective analysis of 28 non-small cell lung cancer operations with the goal of achieving R0 chest wall resection. They compared performance using CT images on a screen with performance using a 3D rendered CT in VR. Overall, VR produced statistically significant more accurate chest wall resection planning predictions and statistically significant better fitting chest wall substitutes compared to CT. In another retrospective review by Deng et al.,^31^ three surgeons devised a preoperative plan based on 3D echocardiography data displayed on a screen (15 cases, three per surgeon) and then reconsidered their approaches after viewing the same data in VR. This intervention led to a modification of the original plan in 60% of cases (9 of 15).

#### 3.6.2 Retrospective reviews on surgical outcomes

A retrospective review conducted by Steineke and Barbery^32^ evaluated the surgical outcomes of microsurgical clipping of middle cerebral artery aneurysms. One group (n=11) was operated based on planning with multiplanar reformations assessed on a conventional monitor, whereas the other group (n=10) was operated on after planning with VR renderings of the same data. The mean case complexity scores of the cases were 2.45 for the CT and DSA group and 2.3 for the VR group, indicating no significant difference in case complexity. However, the mean procedure time was statistically significantly shorter for the VR group compared to the CT and DSA group by an average reduction of 80 min.

#### 3.6.3 Prospective studies on preoperative planning changes

The use of VR following standard surgical planning represents a recurring study design implemented in several selected articles. This approach involves the following steps: standard visualization modalities that predominantly exhibit multiplanar reformations of CT or MRI data, serving as a reference in the planning process. Then the same data are rendered in VR and used for a re-examination of the case. Decisions are revisited and modified if needed.

In a prospective observational pilot study by Sadeghi et al.,^33^ the surgical planning for the lung segmentectomy of 10 patients was initially performed based on CT scans viewed on a conventional monitor. These plans were then re-evaluated after examining the segmented anatomy in VR. The study found that in 40% of cases, the target segments were modified. Three cases saw an extension of the segmentectomy, and in one case, the target segment was completely changed, leading to a successful segmentectomy. Similarly, a prospective observational study conducted by Bakhuis et al.^34^ examined both the changes in surgical planning and subsequent surgical outcomes. It included 50 patients with an indication for pulmonary segmentectomy. Multiplanar reconstructions of CT data were used to create an initial procedure plan. Subsequently, the CT data were rendered in VR and reassessed. This resulted in an adjustment of the surgical plan in 52% of cases. Localization of the tumor in a different segment occurred in 14% of cases, whereas 10% of cases involved a decision for more lung sparing resection, and 28% required an extended segmentectomy, including 1 lobectomy. Pathological examination confirmed radical resection in 98% of patients. Bakhuis et al.^34^ thereby presented a quantitative analysis, focusing on both surgical planning modifications and subsequent surgical outcomes.

Another study employing a similar design was conducted by Ruyra et al.,^35^ investigating transcatheter aortic valve replacement. The initial planning was based on echocardiography, angiography, and CT. In a subsequent step, the same team reassessed the situation in VR. In 45% of the cases, the implant strategy was modified, and one case referred to surgical replacement instead. Similarly, Abjigitova et al.^36^ employed the same study design for ascending aortic surgery. The surgical plan was initially devised after reviewing CT scans and was then re-evaluated in VR. In 33% of cases, the decision was adjusted (n=6).

Furthermore, two studies simply added VR to the planning procedure that used established imaging techniques. Consequently, the impact of VR on surgical decision-making is more difficult to distinguish from other visualization modalities. In the field of neurosurgery, Louis et al.^37^ integrated VR in the standard planning procedure, making it difficult to isolate any specific changes in the surgical plan attributable to VR. The surgeons indicated that VR may have influenced the surgical decision in only two of 49 cases. Extending this line of research, Anthony et al.^38^ presented five complex neurosurgical cases where the use of VR resulted in changes to the operation plan for two out of five cases. The authors illustrated in detail how VR consultation led to these planning modifications.

#### 3.6.4 Prospective studies on surgical outcomes

Staubli et al.^39^ conducted a study comparing two groups of trainees who performed minimally invasive cholecystectomy, using the global operative assessment of laparoscopic skills (GOALS)^40^ as an assessment tool. The GOALS tool is a reliable and validated outcome measure to compare the effect of different training strategies on laparoscopy skill assessed intraoperatively. One group (n=8) prepared in VR, and the other group relied on conventional training methods using conventional monitors (n=5). The trainees in each group were rated by a supervising surgeon, with the VR group receiving a mean score of 16 and the conventional monitor group receiving a mean of 11. Self-assessment scores were 17.5 and 16 for the VR and conventional monitor group, respectively. However, the group differences were not statistically significant.

#### 3.6.5 Case studies and case series

The identified single cases and case series^38^^,^^41^^–^^49^ have methodological limitations as no objective causal inferences can be derived from them. Nevertheless, their authors acknowledged the positive impact of VR in facilitating preoperative planning. Notably, these reports shared a common characteristic of analyzing complex procedures and cases.

For instance, Ramaswamy et al.^42^ presented a case in which a 11-year-old boy required a left ventricular assist device. Conventional imaging alone provided limited insight into whether the implant would fit into the chest and potentially impinge on the mitral valve. Through the application of VR, the researchers were able to position and rule out any impingement on the mitral valve. In another study by Pelizzo et al.,^50^ VR was employed in the preparation of complex congenital lung malformation surgeries in three cases. Based on the VR assessment, the surgeon could more effectively anticipate potential risks. In another case series reported by Romero Lara et al.,^49^ the use of VR successfully supported a complicated case that required a change of tracheal trajectory, slide tracheoplasty, and vascular plexus. The team highlighted a better understanding of specific anatomical details in VR and pointed out that the use of VR stimulated discussions about the challenges posed by the case.

In their study, Anthony et al.^38^ presented a case that explored the application of VR in preoperative planning for complex neurosurgical cases, particularly emphasizing its value in understanding the spatial relationship between vascular pathologies and critical structures. This enhanced spatial comprehension facilitated the surgical plans. Peek et al.^43^ documented a complex case involving forequarter amputation with chest wall resection, wherein VR played a central role in the preoperative planning process. The multidisciplinary surgical team found VR to be a valuable tool for gaining a better understanding of complex anatomical characteristics. However, during the intraoperative phase, it was observed that neither the CT images nor the VR representation perfectly aligned with the actual patient anatomy. The authors speculated that this discrepancy could be attributed to the stretching of the patient’s arm away from the body during the operation, whereas the CT imaging portrayed the arm positioned alongside the body, potentially causing a shift in the anticipated location of the tumor.

#### 3.6.6 Comparative studies

El Beheiry et al.^51^ conducted a study involving practicing and resident surgeons (n=9 for each group) to evaluate the speed and accuracy of breast cancer tumor localization. They analyzed 27 cases, including two healthy control cases, and compared the performance of viewing slice-based reformations of MRI data on a desktop monitor with viewing volumetric renderings of the MRI data in VR. The study found that the performance in VR was significantly faster and more accurate in identifying which breast contained the lesions for both groups. However, there was no significant difference in the number of lesions identified. The accuracy of quadrant determination improved statistically significant for practicing surgeons through VR, but this improvement was not observed among residents. In a similar study by Bakhuis et al.,^36^ the segment assignment for congenital lung abnormalities was compared between slice-based CT viewed on a conventional monitor and VR in five asymptomatic cases. Assigning specific lung segments matched in only one case for two specialists using CT, but this agreement increased to three cases when using VR.

Huettl et al.^52^ conducted a study comparing 3D-printed models and 3D PDFs with VR to identify liver segments in 20 cases, involving students, residents, fellows, and hepatopancreatobiliary experts. The results showed that VR and 3D-printed models significantly outperformed 3D PDFs in identifying the correct segments. Tumor assignment was significantly faster with 3D-printed models compared to VR and 3D PDFs. Additionally, 73% of the participants preferred VR (n=30). A controlled randomized trial described by Santa-Barbara et al.^53^ compared the classification accuracy of proximal humeral fractures using VR with 3D-printed models. Although 3D-printed models performed slightly better, the difference was not statistically significant.

## 4 Discussion

### 4.1 Conceptual Clarity and Visualization Modalities

This review focused on VR presented through an HMD. It is possible to create VR using different systems, such as 3D monitors^54^ or cave automatic virtual reality environment systems,^55^ among others. The selected literature often did not specify the visualization modality that was used to display the datasets. Some studies used the term VR to describe 3D models presented on conventional monitors^56^ or smartphone screens.^57^ For illustration, one study stated that surgeons “reviewed the 3D VR models individually or with their surgical team via a mobile application developed by the sponsor and installed on their smartphones.”^57^ Many articles did not report on the technical setups and specifications of the VR visualization modalities that were used. This impedes the comparison between different imaging modalities of VR. Authors should address this ambiguity, by giving a more precise definition of the used visualization modality. This will ensure consistency, improve comparability, and avoid potential misunderstanding.

We observed that in VR, medical images always appeared in a 3D format and were most often compared to medical images in reformatted planes on conventional monitors. This raises a methodological question: is this a fair comparison? The use of 3D models in VR and the use of 2D images for screens could potentially introduce an artificial bias in favor of VR. An alternative approach could be to compare VR images to 3D images on conventional monitors providing similar interaction and manipulation capabilities. We think that a comparison of 2D images on conventional monitors in the form of reformatted planes (axial/sagittal/coronal) and 3D images in VR is nevertheless a viable way to assess the difference between the visualization modalities. This approach corresponds with the scope of most studies that seek to understand the differences between the standard of care and the new visualization modality of VR.

### 4.2 Technical Specifications

The reporting of technical equipment was often inconsistent and neglected important information. In 30% of the reviewed studies, the type of HMD was not specified. Technical specifications, such as the field of view and the resolution of HMDs, vary widely. The HTC Vive^58^ has a resolution of 1080×1200  pixels per eye, whereas the Meta Quest 2^59^ has a resolution of 1832×1920  pixels per eye. Using different HMDs is likely to result in different outcomes as display quality, tracking performance and usability vary. Authors should specify which HMD was used to enable reproduction and to provide context information.

In 33 out of 52 cases, the literature did not indicate whether HMDs were tethered to a computer and if they provided graphic processing. Even when the information was provided, important details, such as information on VRAM, RAM, and processor, were missing. This information is useful to indicate image quality and frame rate of VR images. A rate of 120 frames per second and more is desirable to prevent simulator sickness.^60^ Lower frame rates force users to adjust their behavior when manipulating images.

### 4.3 Imaging Techniques

Regarding the reporting of medical imaging data used to generate the VR models, it is noteworthy that only 6 out of 35 studies using CT reported the parameters of CT image acquisition and processing. Acquisition and processing parameters of CT/MRI data, such as reconstructive interval, affect 3D model resolution and quality.^61^^,^^62^ The indication of imaging parameters relevant to 3D model quality should be reported when medical imaging data are visualized in VR.

**Fig. 7:**
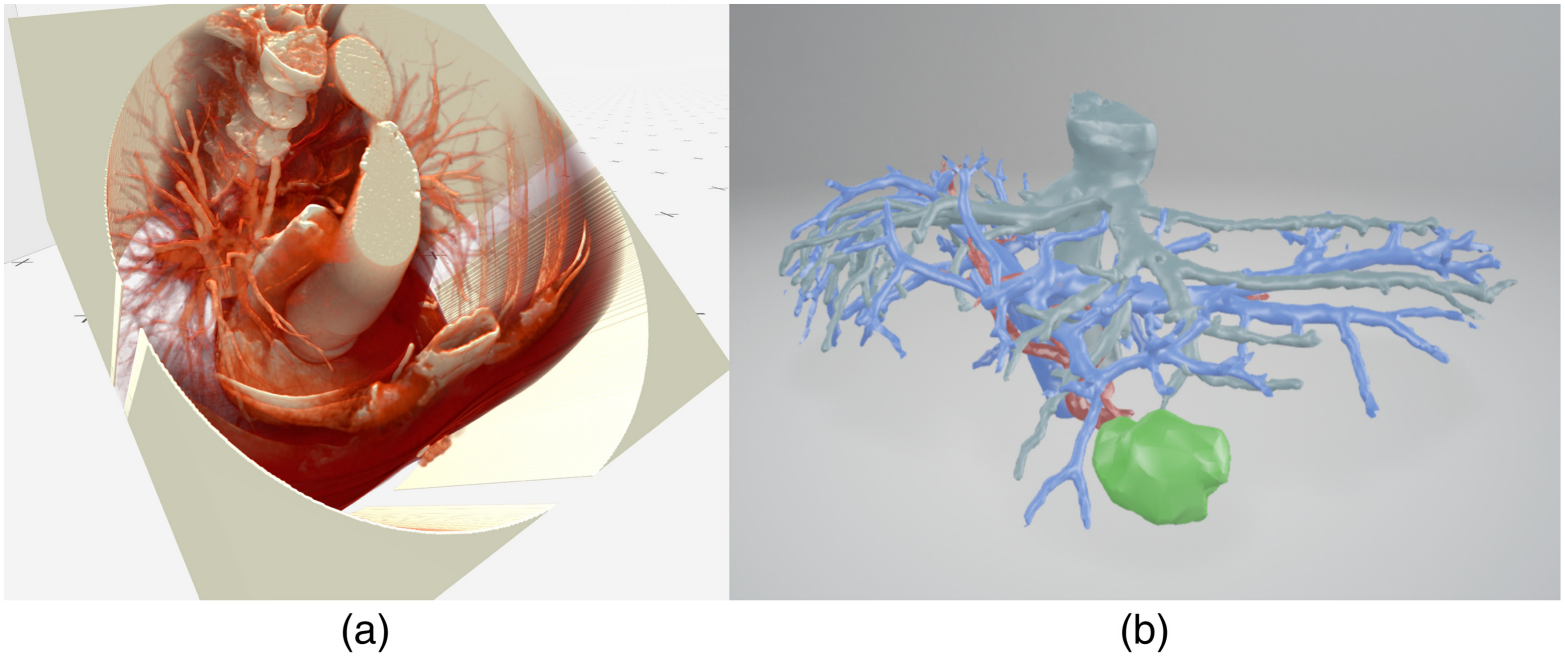
Examples of rendering techniques: (a) volumetric rendering^4^ and (b) mesh.^63^

Another technique that determines the planning decision is the rendering method. Some studies rely on polygon meshes, some on volumetric rendering, and others on both. The distribution of the three types is approximately equal. Most studies did not indicate the rendering method, although they have a substantial impact on what the imaging data show. A polygon mesh is a network of interconnected polygons, typically triangles or quadrilaterals, which collectively form the detailed surface of a three-dimensional object [Fig. 7(b)]. The complexity and realism of this object are dependent on the number of polygons used. Polygon meshes achieve high accuracy through precise segmentation, with surface transparency being pivotal for visualizing internal structures.

On the other hand, volumetric rendering uses a transfer function, which applies variable coloring and opacity levels throughout the structures of a volume [Fig. 7(a)]. This volume is composed of voxels, which are the 3D equivalent of pixels. Appropriately chosen transfer functions can offer a comprehensive view of the entire medical imaging dataset, revealing both surface and internal details. Polygon meshes tend to be less computationally intense, they can be generated manually, semiautomatically, or fully automatically allowing for customization of color and transparency values. Volumetric rendering can enhance the accuracy of medical images by capturing the density and attributes within three-dimensional data. Its accuracy largely hinges on the applied transfer functions, which include color mapping and opacity adjustments, mapping data values to visual properties across a spectrum as well as supplementary algorithms that enhance the rendering.

### 4.4 Segmentation

In 27 out of 41 cases, the type of segmentation was not explicitly stated. The selection of rendering technique often hinges on the type of segmentation performed on the imaging data. The segmentation strategy plays an important role in medical imaging and directly influences the information derived from visualizations. Therefore, reporting on the segmentation method is essential. This includes specifying whether the segmentation was manual, semiautomatic, or fully automatic. A full segmentation of all surgically relevant structures is typically going to be a mesh. When segmentation is confined to specific targets, such as tumors, most studies combine mesh and volumetric rendering of the surrounding relevant structures. Particularly, when using volumetric rendering, reporting on the details of the process is critical, such as the types of presets and algorithms, which were used for the rendering. Furthermore, understanding the amount of additional labor invested in generating the visualization needs to be considered. A detailed reporting can provide insights into the accuracy, reliability, and reproducibility of the visualizations in surgical planning contexts. It furthermore allows to draw conclusions on the total amount of work required, which is important to understand for the integration into clinical workflows.

### 4.5 Software

The functions and scope of software solutions vary widely. In 52 studies, 32 different software solutions were used. They offer different modes of interaction and manipulation and are often tailored for one surgical indication. The large number of custom-made software solutions hinders comparability among the studies. In many cases, the tools to manipulate the VR images were not systematically indicated. This is necessary as the specifications of software impact the individual outcome. Among the most mentioned features are plane cutting and the ability to display the 3D image alongside the 2D counterpart. Direct comparison with 2D images improves assurance of clinicians accustomed to conventional imaging formats, subsequently fostering adoption.^31^

### 4.6 Outcomes by Study Type

Retrospective studies on preoperative surgical planning identified advantages of VR compared to other visualization modalities.^26^^,^^29^^–^^31^ They found that actual surgery improved,^29^ significantly better resection planning predictions and significantly better fitting chest wall substitutes were made^26^ after additional VR review and that substantial plan modifications were made in 57%^30^ of cases and 60%^31^ of cases. Retrospective studies proved to be an effective study type to analyze the use of VR in surgery as ground truth may be established and surgical decisions can be compared with each other. Moreover, there is no risk of compromising treatment standards, and it is easier to gather a large number of cases. A retrospective review^32^ found a statistically significant reduction of surgical procedure time by 80 min on average for the surgeries planned in VR. This is an impressive result, although limited by the number of cases (21). It would be important to replicate this result as it makes a strong case for the use of VR.

Prospective studies using VR to improve decision-making in surgical procedures subsequent to the consultation of initial conventional monitor setups^33^^–^^36^ observed that the surgery plan was adjusted in 33%,^36^ 40%,^33^ 45%,^35^ and 52%^64^ of cases. These adjustments improved the quality of the surgical plans.^33^^,^^34^ These figures represent high rates of surgical plan changes and emphasize the capability of VR to support surgical decision making. Staubli et al.^39^ found advantages of VR over reformatted slice-based image modalities on conventional monitor setups. Notably, they engaged surgical trainees in performing surgeries using VR exclusively as the preparatory tool. Within this context, a marginal advantage of VR over conventional monitors was observed, albeit without achieving statistical significance in terms of surgical performance measured by the GOALS metric.^40^ These findings are at least a promising result, given that surgical trainees have typically spent more planning time with conventional monitors than with VR. They indicate that performance loss due to VR seems unlikely. It must be noted however that in the study no 3D views were provided on the conventional monitor, which is already a key difference, potentially introducing a bias in favor of VR.

Regarding the reviewed case studies and case series,^38^^,^^41^^–^^49^ we noticed that the indications were mostly highly complex cases with high risks of complications. These surgeries require a high level of spatial understanding and precision due to the critical nature of the target structure and its surroundings. Initial evidence supporting the use of VR in such cases has been presented in a study conducted by Steineke and Barbery^32^ showing that VR statistically significantly decreased procedure time in microsurgical clipping of middle cerebral artery aneurysms. This finding highlights the importance of conducting controlled studies with objective outcomes. Apparently, VR may be used as an additional tool alongside standard imaging to enhance patient safety while maintaining the current standards of care.

The findings from comparative studies^34^^,^^51^^–^^53^ offer encouraging outcomes, substantiating the efficiency of VR in contrast to other visualization modalities. One study found no statistically significant difference between VR and 3D printed models for the classification accuracy of fractures,^53^ but stated that 3D printing is more expensive and requires additional time.^65^ Another study found VR to be comparable to 3D printed models but statistically significantly better than the use of a conventional monitor setup for segment assignment of liver tumors.^52^ Two studies reported better performance,^51^ faster performance^51^ and higher agreement among evaluators^34^ in performing segment identification for tumors and lesions compared against other visualization methods. These promising results are particularly noteworthy, as they highlight the advantages attributed to VR within a context that permits direct comparison of outcomes to ground truth. Regarding surgical planning and surgical outcomes, the results of the reviewed studies strongly support a positive impact of VR on preoperative surgical decision making and surgical outcomes. Still, a systematic quantitative analysis such as a meta-analysis would be difficult to execute as the outcome data in the reviewed studies is very diverse and often subjective in nature.

### 4.7 Comparison with Previous Outcomes

Lan et al.^11^ provided a systematic review on surgical planning in VR with similar inclusion criteria to this review. Since their cut-off date aligns precisely with our start date, there is no overlap in the studies. Our results substantiate their claim that study designs and outcomes are heterogeneous, hindering comprehensive meta-analyses. The most compelling evidence would stem from clinical outcome data comparing surgeries planned in VR to other visualization modalities. It is evident that most planning decisions were not contested in actual surgeries. Instead, studies in both reviews relied on other variables to assess anatomical understanding and the quality of surgical decision-making. In accordance with Lan et al., we found that most results of the studies favored VR; however, the outcomes remain specific, artificial, and isolated. Consequently, we agree with Lan et al. that future VR research needs to shift toward high-quality studies that measure comparable clinical outcomes for patients and comparable ergonomic outcomes for surgeons.

## 5 Conclusion

The past couple of years have witnessed a remarkable increase in the number of studies focusing on VR applications for surgical planning. The interest in VR has substantially extended the amount of use cases, indications, devices, and applications that are considered suitable for surgical planning in VR. The outcomes of the reviewed studies have consistently demonstrated a positive impact on the quality of surgical decision-making. Several outcomes support this claim: a key finding is that surgical planning in VR leads to substantial changes in surgical plans if it follows up on planning with other visualization modalities. Evidence from isolated studies discover some benefits for operation time (significant), surgical performance measured by standardized tools (trend) and planning time (significant) if image preparation time is excluded. Some results suggest that VR improves decision making in complex or difficult cases, such as the identification of potential bottlenecks and risks of the forthcoming surgery. As these conclusions are drawn from single-case studies, they lack statistical evidence and are mostly drawn from subjective assumptions. Some results suggest that VR-based surgical planning improves the localization and spatial comprehension of pathological changes, when compared to other imaging modalities, supported by objective outcomes.^26^^,^^51^^,^^52^ However, this claim is not yet sufficiently tested and requires further backing.

Altogether, a coherent analysis of the studies is difficult to draw. This has different reasons: study designs and methods vary widely; many studies lack detailed information on technical aspects. The compared visualization modalities are diverse, and the surgical procedures differ substantially. This makes the outcomes hardly comparable. Accordingly, the reporting on methods and technical implementation needs to be improved to enable replication and enhance clarity. We have identified four areas that require further research to strengthen the evaluation of VR in surgical planning.

### 5.1 Improving Study Designs

The findings indicate that HMD-based VR research for preoperative surgical planning is still in the early stages of exploring the capabilities of the medium. The field should produce more studies with broader evidence, such as prospective, randomized and controlled multicenter studies focusing on objective clinical outcomes with adequate amounts of participants and cases. This would enable clinical recommendations on the use of VR. Moreover, there should be more comprehensive and detailed reporting on technical implementation to enhance reproducibility and to support clinicians in adoption. We recommend that a comprehensive reporting should encompass details of the employed HMDs and how it was used (standalone and tethered), the software application along with its deployed manipulation tools, specifications of the tethered computer (including RAM, VRAM, and processor specifications), relevant medical imaging parameters, as well as explicit indication of the segmentation and rendering methodologies. Ideally, the frame rate during the use of the HMD should also be included.

### 5.2 Enhancing Visual Knowledge and Technical Proficiency

Physicians are trained to study cross sectional or “sliced” images one after another, to render them cognitively, and to ascribe them to the three-dimensional body of a patient during an intervention. 3D models used in VR with HMDs challenge this well-established visual paradigm. The interpretation of VR images requires learning new skills and to work with new tools, including operations such as zoom, rotate, tilt, and navigate in 3D, which will demand training. Although conventional input devices, such as mouse and keyboard, are considered familiar but less intuitive, manipulating and navigating through VR images requires technical skill. This visual knowledge should be considered as a prerequisite for applying VR in surgical planning. In the long run, this knowledge needs to complement the existing skills to interpret cross-sectional images. We expect that additional training for staff will be needed. Consequently, further research needs to address the visual and technical proficiency that physicians need to acquire to cope with this new architecture of image display. This may also positively affect the lack of conceptual clarity of the notion of VR that was found in some research papers. Most importantly, the improved technical proficiency will be a strong driver in the adoption of VR into clinical practices where reliability and efficiency are a must.

### 5.3 Implementation of VR into Clinical Settings and Workflows

Integrating VR-based planning into clinical routines was described as one of the major challenges and requires further research. This includes the improvement of collaboration and teamwork as well as technical solutions, such as cloud rendering, integration with hospital information systems, and data interoperability standards. Cloud rendering is a neglected topic to be considered in future research since it facilitates the implementation process and enables the use of sophisticated rendering and segmentation algorithms. Notably, no studies highlighted substantial implementation challenges stemming from simulator sickness.

### 5.4 Improving VR Software Usability and Design

The majority of the assessed research articles investigate if VR can support physicians in surgical planning, yet they do not investigate the mechanisms through which VR facilitates this support. Future studies should explore the use of tools for interacting with VR images. Particularly, interaction design concepts, such as tools for navigation, plane cutting, and image manipulation (such as rotating or zooming), of the medical images in VR should be more systematically evaluated. Enhancing the design and usability of VR software is crucial for leveraging its potential.^66^ Design strategies, including the “look and feel” of VR need to adapt to the new visualization modality encompassing visual parameters, such as color, contrast, texture, contour, lighting, or motion. Interaction possibilities, such as eye-tracking and hand-tracking, are evolving and are being integrated into developer frameworks. The automation of image segmentation using AI techniques has the potential to facilitate image manipulation within VR software in the future, particularly with regard to segmentation.^33^^,^^64^ The application of artificial intelligence (AI) in the form of convolutional neural networks is currently changing the way segmentation of medical images is conducted.^67^ Some researchers are asking for deep-learning models parallel to the large language models of today but for medical image tasks, such as segmentation.^68^ There is a domain-specific, open-source AI framework used by over 1 million researchers for medical image segmentation with a focus on 3D imaging accelerating the development of AI for 3D medical image segmentation.^69^ AI is time efficient and largely accurate when segmenting, enabling the efficient generation of accurate 3D models and thereby enabling VR. However, legal and ethical considerations as well as clinical standards will have to be revisited to make use in clinical practice feasible. Due to advancing technology and the democratization of VR that takes place right now, opportunities for the effective and efficient use of VR in surgical planning becomes more economically viable and plausible even in the short term.

## Supplementary Material



## Data Availability

The research data for this paper consist of Tables S1 and S2 and can be accessed in the Supplementary Material.
