# Supplementary material for: Surgical planning in virtual reality: a systematic review
Source: J Med Imaging (Bellingham). 2024 Apr 25;11(6):062603. doi: 10.1117/1.JMI.11.6.062603 (PMC11043584; doi:10.1117/1.JMI.11.6.062603)
Supplement: Supplementary file 1 [file JMI_011_062603_SD001.pdf]

Table S1 Databases and Search Queries

Database	Search Query	Searched in	Additional filters	Number of results
ACM	[[Abstract: vr] OR [Abstract: virtual reality] OR [Abstract: virtual-reality]] AND [[Abstract: surgery] OR [Abstract: operation] OR [Abstract: surgical]] AND [[Abstract: planning] OR [Abstract: pre-operative] OR [Abstract: preoperative] OR [Abstract: pre-op] OR [Abstract: presurgical] OR [Abstract: pre-surgical] OR [Abstract: preplanning] OR [Abstract: pre-planning]] AND [E-Publication Date: (04/01/2021 TO 05/31/2023)]	Abstract	04-01-2021 to 10-05-2023	221
Cochrane CENTRAL	VR OR virtual reality OR virtual-reality in Title Abstract Keyword AND surgery OR operation OR surgical in Title Abstract Keyword AND planning OR pre-operativ OR preoperative OR pre-op OR presurgical OR pre-surgical OR preplanning OR pre-planning in Title Abstract Keyword - (Word variations have been searched)	Title + Abstract	04-01-2021 to 10-05-2023	145
Embase (through Ovid)	((VR or virtual reality or virtual-reality) and (surgery or operation or surgical) and (planning or pre-operative or preoperative or pre-op or presurgical or pre-surgical or preplanning or pre-planning)).ab.	Abstract	2021 to 2023	304
Google Scholar	allintitle: virtual reality "pre operative" OR "pre-operative" OR preoperative OR planning OR surgery OR surgical	Title	2021 to 2023	483
IEEE Xplore	("All Metadata":VR OR "All Metadata":virtual reality OR "All Metadata":virtual-reality) AND ("All Metadata":surgery OR "All Metadata":operation OR "All Metadata":surgical) AND ("All Metadata":planning OR "All Metadata":pre-operativ OR "All Metadata":preoperative OR "All Metadata":pre-op OR "All Metadata":presurgical OR "All Metadata":pre-surgical OR "All Metadata":preplanning OR "All Metadata":pre-planning)	All Metadata	2021 to 2023	98
PubMed	((VR[Title/Abstract] OR virtual reality[Title/Abstract] OR virtual-reality[Title/Abstract]) AND (surgery[Title/Abstract] OR operation[Title/Abstract] OR surgical [Title/Abstract])) AND (planning[Title/Abstract] OR pre-operative[Title/Abstract] OR preoperative[Title/Abstract] OR pre-op[Title/Abstract] OR presurgical[Title/Abstract] OR pre-surgical[Title/Abstract] OR preplanning[Title/Abstract] OR pre-planning[Title/Abstract])	Title + Abstract	04-01-2021 to 10-05-2023	253
Web of Science Core Collection	Results for VR OR virtual reality OR virtual-reality (Abstract) AND surgery OR operation OR surgical (Abstract) AND planning OR pre-operativ OR preoperative OR pre-op OR presurgical OR pre-surgical OR preplanning OR pre-planning (Abstract)	Abstract	04-01-2021 to 10-05-2023	309
Total				1813

**Table S2**	Sorted by year of publication																	
**Title**	**Authors**	**Year of publication**	**Study design**	**Surgical discipline**	**Procedure / indication**	**Name of VR-software**	**Head-mounted display**	**Rendering technique**	**Segmentation**	**Computer**	**Imaging input for VR model**	**Visualization modalities, comparisons (versus) and presentation order (then)**	**Participants**	**No. of cases**	**Outcome measures**	**Comparison of VR with...**	**Favours VR**
Patient-specific virtual reality technology for complex neurosurgical cases: illustrative cases	Anthony et al.	2021	case series	neurosurgery	spinal cord cavernoma, clinoidal meningioma, anaplastic oligodendroglioma, giant aneurysm, and arteriovenous malformation	Surgical Theater	not reported	Volumetric + Meshes	Threshold setting + (Segmentation?)	no info	CT + MRI	monitor (2D CT/MRI) + VR	not reported	5	surgical planning (subjective)	monitor (2D CT/MRI) alone	yes
Preoperative planning using virtual reality for percutaneous transseptal valve-in-valve transcatheter mitral valve replacement: a case report	Castellanos et al.	2021	case report	interventional cardiology	percutaneous transseptal valve-in-valve transcatheter mitral valve replacement	Surgical Theater	Oculus Rift S	Volumetric	Segmented	no info	CT (0.4 mm)	monitor (2D echo/CT) + VR	not reported	1	surgical planning (subjective)	none	yes
Breast Magnetic Resonance Image Analysis for Surgeons Using Virtual Reality: A Comparative Study	El Beheiry et al.	2021	experiment	general surgery	breast cancer	DIVA	HTC Vive	Volumetric	No	no info	MRI	VR versus monitor (2D MRI)	18 breast surgeons (nine residents and nine practicing surgeons)	25	analysis time, accuraccy of determination of which breatst contained lesion, determination of the number of lesions, accuracy of quadrant determination	monitor (2D MRI)	yes
Investigating the utility of VR for spatial understanding in surgical planning: evaluation of head-mounted to desktop display	Hattab et al.	2021	experiment	general surgery	liver tumor	adapted version of IMHOTEP framework	HTC Vive	Meshes	Manual Segmentation	NVIDIA GeForce GTX 1080 graphics cards and Intel i7-6700K 4.0 GHz processor	CT	VR + 3D printed model versus monitor (2D CT)	56 (medical staff and students)	1	understanding, direction estimation, usability	monitor (2D CT)	equal
Virtual reality and 3D printing improve preoperative visualization of 3D liver reconstructions—results from a preclinical comparison of presentation modalities and user's preference	Huettl et al.	2021	experiment	general surgery	identification of liver tumor	custom made	HTC Vive	Meshes	Segmented	MSI Gaming GT63 (Micro-Star int'l Co., Ltd., New Taipei City 235, Taiwan)	CT/MRI	VR versus 3D printed model versus monitor (3D CT/MRI)	30 (medical students (n = 10), residents (n = 10), fellows (n = 5) and hepatopancreatobiliary experts (n = 5))	20	1) segment identification , 1) tumor assigment, 3) usability and user experience	3D printed model, monitor (3D CT/MRI)	1) yes, 2) no, 3) yes
Virtual Reality (VR) Simulation and Augmented Reality (AR) Navigation in Orthognathic Surgery: A Case Report	Jo et al.	2021	case report	oral and maxillofacial surgery	orthognathic surgery	not reported	HTC Vive Pro	Meshes	Segmented	no info	CT + facial scan	VR	not reported	1	avg. difference preplanned data and post-operative results, surgical planning (subjective)	none	yes
Multidisciplinary Virtual Three-Dimensional Planning of a Forequarter Amputation With Chest Wall Resection	Peek et al.	2021	case report	thoracic surgery	forequarter amputation with chest wall resection	MedicalVR	not reported	Volumetric + Meshes	Segmented	no info	CT	monitor (2D CT) + VR	not reported	1	surgical planning (subjective)	none	yes
Developing Virtual Reality Head Mounted Display (HMD) Set-Up for Thoracoscopic Surgery of Complex Congenital Lung Malformations in Children	Pelizzo et al.	2021	case series	thoracic surgery	complex congenital lung malformations	custom made	Oculus Quest 1	Meshes	Semiautomatic Segmentation	no info	CT (0.6 mm)	monitor (CT/MRI) +VR	1 experienced surgeon	3	surgical planning (subjective)	none	yes

**Table S2**	Sorted by year of publication																
**Title**	**Authors**	**Year of publication**	**Study design**	**Surgical discipline**	**Procedure / indication**	**Name of VR-software**	**Head-mounted display**	**Rendering technique**	**Segmentation**	**Computer**	**Imaging input for VR model**	**Visualization modalities, comparisons (versus) and presentation order (then)**	**Participants**	**No. of cases**	**Outcome measures**	**Comparison of VR with...**	**Favours VR**
Virtual reality–based 3-dimensional localization of stereotactic EEG (SEEG) depth electrodes and related brain anatomy in pediatric epilepsy surgery	Phan et al.	2021	case series	neurosurgery	localization of stereotactic EEG depth electrodes and related brain anatomy in pediatric epilepsy surgery	Surgical Theater	HTC Vive	Volumetric + Meshes	Semiautomatic Segmentation	no info	CT + MRI	VR + monitor (2D CT/MRI/SEEG)	1 neurosurgeon	20	VR demand and usage, surgical planning (subjective)	monitor (2D CT/MRI/SEEG)	yes
Fast-track virtual reality for cardiac imaging in congenital heart disease	Raimondi et al.	2021	experiment	cardiothoracic surgery	complex congenital heart disease	DIVA	not reported	Volumetric	No	no info	MRI	VR versus 3D printed model versus monitor (3D MRI)	senior pediatric heart surgeon	3	post-processing time, point rating on visualization, surgical planning (subjective)	3D printed model, monitor (3D MRI)	yes
Virtual reality-guided left ventricular assist device implantation in pediatric patient: Valuable presurgical tool	Ramaswamy et al.	2021	case report	cardiothoracic surgery	left ventricular assist device implantation / optimal positioning of the left ventricular assist device	custom made	Oculus Rift	Meshes	Segmented	no info	CT	monitor (2CT) + monitor (2D Echo) + VR	not reported	1	surgical planning (subjective)	none	yes
Virtual Reality for Surgical Planning - Evaluation Based on Two Liver Tumor Resections	Reinschluessel et al.	2021	case series	general surgery	liver tumor resections	custom made	HTC Vive Pro	Meshes	Manual Segmentation	Intel i7 processor and an Nvidia 2080 TI graphics card	MRI	monitor (2D MRI) + VR	4 surgeons	2	planning time in VR, usability, surgical planning (subjective)	none	yes
Use of Virtual Reality and Three-Dimensional Printing in the Surgical Planning of Slide Tracheoplasty	Romero Lara et al.	2021	case series	thoracic surgery	slide tracheoplasty	custom made	HTC Vive	Meshes	Segmented	no info	CT (0.5 mm)	1) monitor (2D CT) 2) monitor (2D CT) + 3D printed model 3) monitor (2D/3D CT + VR	not reported	3	post-processing time, surgical planning (subjective)	monitor (2D CT), monitor (2D CT) + 3D printed model, monitor (2D/3D) CT	yes
Immersive Virtual Reality Heart Models for Planning of Transcatheter Paravalvular Leak Closure: A Feasibility Study	Sadeghi et al.	2021	case series	interventional cardiology	transcatheter paravalvular leakage closure by means of a closure device	MedicalVR	not reported	Meshes	Segmented	no info	CT	monitor (3D echocardiogram) versus monitor (2D CT) versus monitor (3D CT) versus VR	not reported	6	plug approximation compared to final plug	monitor (3D echocardiogram), monitor (2D CT), monitor (3D CT)	no
Magnetic resonance cholangiopancreatography enhanced by virtual reality as a novel tool to improve the understanding of biliary anatomy and the teaching of surgical trainees	Staubli et al.	2021	1) usability study 2) experiment 3) randomized controlled trial	general surgery	minimally invasive cholecystectomy	Specto VR	HTC Vive	Volumetric	No	ASUS ROG Zephyrus GX501GI-EI005T (15.60″; full HD; Intel Core i7-8750H, 16GB; 512GB hard-drive; and graphics processing unit Nvidia GTX1080 MaxQ)	MRI	1) VR 2) VR versus monitor (2D MRI) 3) monitor (2D MRI) versus VR	1) 34 (doctors from all specialties and with all experience levels); 2) 27 (doctors from all specialties and with all experience levels); 3) 13 (surgical trainees)	1) ? 2) 15 3) 13	1) usability (SSQ), 2) recognition of anatomic structures (time, correctness), 3) surgical outcomes (GOALS score)	1) none 2) monitor (2D MRI) 3) monitor (2D MRI)	1) Yes, 2) Yes, 3) Yes

**Table S2**	Sorted by year of publication																
**Title**	**Authors**	**Year of publication**	**Study design**	**Surgical discipline**	**Procedure / indication**	**Name of VR-software**	**Head-mounted display**	**Rendering technique**	**Segmentation**	**Computer**	**Imaging input for VR model**	**Visualization modalities, comparisons (versus) and presentation order (then)**	**Participants**	**No. of cases**	**Outcome measures**	**Comparison of VR with...**	**Favours VR**
Extended reality platform for minimally invasive endoscopic evacuation of deep-seated intracerebral hemorrhage: illustrative case	Steineke and Barbery	2021	case report	neurosurgery	spontaneous intracerebral hemorrhage	Surgical Theater	Oculus Rift	Volumetric + Meshes	Yes (The model displayed the skull, arteries, lateral ventricles, corticospinal tracts (CSTs), and hemorrhage in detail)	no info	CT + MRI	monitor (2D CT/MRI) + VR	1 surgeon	1	surgical planning (subjective)	none	yes
The effect of virtual reality on temporal bone anatomy evaluation and performance	Timonen et al.	2021	experiment	otorhinolaryngology	temporal bone anatomy evaluation	Adesante SurgeryVision	HTC Vive Pro	Volumetric	no	no info	CT (0.4 mm)	monitor (2D CT) versus VR	10 (novice group (n = 5); expert group (n= 5))	5	identification, measurements (error rate, performance time)	monitor (2D CT)	yes
Removal of a foreign body by rigid bronchoscope after virtual reality-aided presurgical planning: A case report	Torii et al.	2021	case report	otorhinolaryngology	removal of a foreign body by rigid bronchoscope	BananaVision	not reported	Volumetric	no info	no info	CT (2.0 mm)	monitor (2D CT) + VR	not reported	1	surgical planning (subjective)	none	yes
Advantages of a Training Course for Surgical Planning in Virtual Reality for Oral and Maxillofacial Surgery: Crossover Study	Ulbrich et al.	2021	experiment	oral and maxillofacial surgery	oral and maxillofacial surgery	Elucis	HTC Vive Pro	Volumetric + Meshes	The task was to segment	same workstation (AMD Ryzen 3900X CPU with 64 GB of memory and an RTX 2080 Ti graphics card).	CT	VR + VR (2D CT) versus monitor (2D CT)	6	26	learning curve, segmentation speed, qualitative result, workload, preference	monitor (2D CT)	yes
Virtual Reality in the Preoperative Planning of Adult Aortic Surgery: A Feasibility Study	Abjigitova et al.	2022	feasibility study	cardiothoracic surgery	adult aortic surgery	MedicalVR	Oculus Rift S	Volumetric + Meshes	Manual Segmentation	no info	CT	first monitor (2D CT), then VR	10 cardiothoracic surgeons	6	surgical planning (changes)	monitor (2D CT) alone	yes
Essential Surgical Plan Modifications After Virtual Reality Planning in 50 Consecutive Segmentectomies	Bakhuis et al.	2022	prospective case series	thoracic surgery	pulmonary segmentectomy	MedicalVR	not reported	Volumetric + Meshes	AI Segmented	no info	CT	first monitor (2D CT), then VR	3 surgeons	50	surgical planning (rate of changes, type of changes), surgical outcome (rate of total resection)	monitor (2D CT) alone	yes
Preoperative visualization of congenital lung abnormalities: hybridizing artificial intelligence and virtual reality	Bakhuis et al.	2022	experiment	thoracic surgery	congential lung abnormalities	MedicalVR	not reported	Volumetric + Meshes	Semiautomatic Segmentation	no info	CT	monitor (2D CT) versus VR	2 specialists (a pedriatic thoracic surgeon and a pedriatic thoracic radiologist, with 15 and 10 years of experience in CLA)	5	surgical planning (assignment of CLA lesion to lung segment, surgical strategy)	monitor (2D CT)	yes
A collaborative virtual reality environment for liver surgery planning	Chheang et al.	2022	software elaboration + user study	general surgery	liver surgery	custom made	HTC Vive	Meshes	Segmented	Intel®CoreTMi7-8700K CPU @3.70 GHz (12 CPUs) processor, an NVIDIA GeForce GTX 1080 (8 GB VRAM) graphics card, and 32 GB RAM	CT	VR	3 surgical residents	not reported	applicability, usability (SUS, IPQ)	none	not applicable

Table S2

Sorted by year of publication

Title	Authors	Year of publication	Study design	Surgical discipline	Procedure / indication	Name of VR-software	Head-mounted display	Rendering technique	Segmentation	Computer	Imaging input for VR model	Visualization modalities, comparisons (versus) and presentation order (then)	Participants	No. of cases	Outcome measures	Comparison of VR with...	Favours VR
Echo-Endoscopy Combined with Virtual Reality: A Whole Perspective of Laparoscopic Common Bile Duct Exploration in Children	Destro et al.	2022	retrospective case series	general surgery	laparoscopic common bile duct exploration	custom made	Oculus Quest 1	Meshes	Semiautomatic Segmentation	no info	MRI	VR + monitor (2D MRI/CT) versus monitor (2D MRI/CT)	not reported	4 in VR (12 total)	surgical planning (subjective)	monitor (2D MRI/CT), (2D MRI/CT)	yes
Virtual reality surgical planning for endoscopic endonasal approaches to the craniovertebral junction	Filimonov et al.	2022	retrospective case series	neurosurgery	endoscopic endonasal craniovertebral junction surgery	Surgical Theater	not reported	Volumetric + Meshes	Manual Segmentation	no info	CT + MRI	monitor (2D CT/MRI) + VR	not reported	5	surgical planning (subjective)	none	yes
Using Virtual Reality for Deep Inferior Epigastric Perforator Flap Preoperative Planning	Freidin et al.	2022	prospective case series	plastic surgery	evaluating the abdominal vascular anatomy before autologous breast reconstruction	3D Systems (3D Systems Inc., Littleton Colo.))	HTC Vive	Volumetric + Meshes	Segmented	no info	CT (0.625 mm)	monitor (2D CT) + VR	not reported	30	surgical planning (rate of changes and rated contribution), similarity of VR to actual physicality	monitor (2D CT)	yes
Clinical 3D modeling to guide pediatric cardiothoracic surgery and intervention using 3D printed anatomic models, computer aided design and virtual reality	Gosh et al.	2022	retrospective review	cardiothoracic surgery	pediatric cardiothoracic surgery	3D Slicer, SlicerVR	Valve Index	Meshes	Semiautomatic Segmentation	standard PC with a RTX 2080TI graphics processor	CT+ MRI	3D printed model + monitor (3D CT/MRI) + monitor (3D (CAD modeling) CT/MRI) + VR	not reported	16	historic demand on different 3D modalities 2018, 2019, 2020	3D printed model, monitor (3D CT/MRI), monitor (3D (CAD modeling) CT/MRI)	not applicable
Study of comparative surgical exposure to the petroclival region using patient-specific, petroclival meningioma virtual reality models	Jean et al.	2022	experiment	neurosurgery	petroclival meningiomas near the central clival depression	Surgical Theater	not reported	Volumetric + Meshes	Segmented	no info	CT + MRI (<1mm)	VR	not reported	15	blinded volume and exposure	none	not applicable
Early Experience With Virtual and Synchronized Augmented Reality Platform for Preoperative Planning and Intraoperative Navigation: A Case Series	Louis et al.	2022	prospective case series	neurosurgery	microsurgical procedures	Surgical Theater	not reported	Volumetric + Meshes	Manual Segmentation	no info	CT + MRI / DTI (<1mm)	VR	7 neurosurgeons (attendings with an average of 3.8 ± 1.6 and 1.8 ± 1.6 yr of experience with VR for surgical planning)	49	use, planning time, surgery outcomes, subjective measures	none	yes
Enhanced 3D visualization for planning biventricular repair of double outlet right ventricle: a pilot study on the advantages of virtual reality	Milano et al.	2022	pilot study	cardiothoracic surgery	complex double outlet right ventricle	custom made	Oculus Rift	Meshes	Segmented	no info	CT + MRI	first monitor (2D CT/MRI), then monitor (3D CT/MRI), then 3D printed model, then VR	2 pedriatic cardiac surgeons	10	surgical planning (accordance with performed operation)	(2D CT/MRI), monitor (3D CT/MRI), 3D printed model	yes
DICOM 3D viewers, virtual reality or 3D printing – a pilot usability study for assessing the preference of orthopedic surgeons	Popescu et al.	2022	pilot study	orthopedic surgery	orthopedic surgery	custom made	HTC Vive	Meshes	Manual Segmentation	no info	CT (1mm)	3D printed model and monitor (2D/3D CT) and VR	15 surgeons (29,3 average years of experience)	not reported	1) usefulness, 2) preference, 3) preferred order of the modalities	monitor (2D/3D CT), 3D printed model	1) No, 2) No, 3) No
Use of virtual reality for procedural planning of transcatheter aortic valve replacement	Ruyra et al.	2022	prospective case series	interventional cardiology	transcatheter aortic valve replacement	VisuaMed	not reported	Volumetric + Meshes	Segmented	no info	CT	first monitor (2D echo/angiography/CT, 3D CT) , then VR	not reported	11	surgical planning (rate of changes)	monitor (2D echo/angiography /CT, 3D CT)	yes

Table S2

Sorted by year of publication

Title	Authors	Year of publication	Study design	Surgical discipline	Procedure / indication	Name of VR-software	Head-mounted display	Rendering technique	Segmentation	Computer	Imaging input for VR model	Visualization modalities, comparisons (versus) and presentation order (then)	Participants	No. of cases	Outcome measures	Comparison of VR with...	Favours VR
Virtual reality and artificial intelligence for 3-dimensional planning of lung segmentectomies	Sadeghi et al.	2022	prospective case series	cardiothoracic surgery	lung segmentectomies	MedicalVR	not reported	Volumetric + Meshes	AI Segmented	no info	CT	first monitor (2D CT), then monitor (2D CT), then VR (grayscale), then VR	2 surgeons	10	surgical planning (rate of changes)	monitor (2D CT)	yes
Preoperative Virtual Reality Surgical Rehearsal of Renal Access during Percutaneous Nephrolithotomy: A Pilot Study	Sainsbury et al.	2022	pilot study	urology	percutaneous nephrolithotomy	Marion K181 PCNL simulator	HTC Vive	Meshes	Segmented	Marion K181 PCNL simulator	CT	monitor (2D CT) + VR + haptic feedback	not reported	1	surgical planning (subjective)	none	yes
New technologies for the classification of proximal humeral fractures: Comparison between Virtual Reality and 3D printed models—a randomised controlled trial	Santa-Barbara et al.	2022	randomized controlled trial	orthopedic surgery	proximal humeral fractures	custom made	Oculus Quest 2	Meshes	Segmented	no info	CT	3D printed model versus VR	37	36 (18 VR group, 18 control group)	rate of correct classification	3D printed model	equal
Microsurgical clipping of middle cerebral artery aneurysms: preoperative planning using virtual reality to reduce procedure time	Steineke and Barbery	2022	retrospective review	neurosurgery	middle cerebral artery aneurysm	Surgical Theater	Oculus Rift	Volumetric + Meshes	No	no info	CT (0.64 mm and DSA scans (0.34mm)	monitor (2D CT + DSA) versus VR	1 expert neurosurgeon attending (13 to 16 years of experience)	21 (control group 11; VR group 10)	procedure time, Charlson Comorbity Index	monitor (2D CT + DSA)	yes
Immersive Three-dimensional Computed Tomography to Plan Chest Wall Resection for Lung Cancer	Thumerel et al.	2022	retrospective review	thoracic surgery	chest wall resection for lung cancer	nonmedical beta test version of software (AW Virtual Reality prototype:	not reported	Volumetric + Meshes	Segmented	no info	CT	VR + VR (2D CT) versus monitor (2D CT)	3 resident surgeons, 5 senior surgeons	28	resection planning predictions, chest wall substitutes predictions	monitor (2D CT)	yes
3D Virtual Reality Imaging of Major Aortopulmonary Collateral Arteries: A Novel Diagnostic Modality	Van de Woestijne et al.	2022	proof-of-concept study	cardiothoracic surgery	repair of major aortopulmonary collateral arteries	MedicalVR	not reported	Volumetric + Meshes	Segmented	no info	CT	monitor (2D CA/CT) + VR	2 surgeons	7	surgical planning (changes)	monitor (2D CA/CT) alone	yes
Three-Dimensional Modeling of Complex Pediatric Intracranial Aneurysmal Malformations With a Virtual Reality System	Yan et al.	2022	retrospective review	neurosurgery	middle cerebral artery aneurysmal malformations	Surgical Theater	Oculus Rift	Volumetric + Meshes	Yes?	no info	CT + MRI	monitor (2D CT/MRI) + VR	1 experienced surgeon	5	surgical planning (subjective)	none	yes

Table S2	Sorted by year of publication																	
**Title**	**Authors**	**Year of publication**	**Study design**	**Surgical discipline**	**Procedure / indication**	**Name of VR-software**	**Head-mounted display**	**Rendering technique**	**Segmentation**	**Computer**	**Imaging input for VR model**	**Visualization modalities, comparisons (versus) and presentation order (then)**	**Participants**	**No. of cases**	**Outcome measures**	**Comparison of VR with...**	**Favours VR**
A Virtual Reality System for Improved Image-Based Planning of Complex Cardiac Procedures	Deng et al.	2023	software elaboration + user study + retrospective case series review	cardiothoracic surgery	complex cardiac surgery	custom made	HTC Vive	Volumetric	No	Dell Alienware laptop with an Intel i9-8950HK 2.90 GHz CPU, 16GB RAM, Nvidia GTX1080 GPU with 8GB RAM or a Dell Alienware desktop with an Intel i7-8700 3.20GHz CPU, 32GB RAM, Nvidia GTX 1080Ti GPU with 11GB RAM.	dynamic 3D echo	1) VR versus monitor (2D echo), 2) VR versus monitor (2D/3D echo), 3) monitor (2D echo data) then VR	1) 13 clinicians (1 trainee, 4 junior and 8 senior) 5 imaging cardiologists, 5 cardiac physiologists, 2 cardiac surgeons and 1 cardiac interventionist 2) 5 cardiologists, (3 imaging cardiologists and 2 physiologists, of whom 4 were senior (5+ years of experience) with 1 junior (<5 years experience)) 3) 5 pedriatic cardiothoracic surgeons (3 with >15 years experience, 1 senior trainee >3 years, 1 junior trainee <3 years)	1) 1, 2) 4, 3) 15	1) usability (preferences), 2) measurements (accuracy in comparison to phantom model), 3) planning (rate of changes)	1) monitor (2D echo), 2) monitor (2D/3D echo), 3) monitor (2D echo data)	yes
Virtual Reality Cardiac Surgical Planning Software (CorFix) for Designing Patient-Specific Vascular Grafts: Development and Pilot Usability Study	Kim et al.	2023	usability study	cardiothoracic surgery	designing patient-specific vascular grafts	CorFix	Oculus Rift S	Meshes	Manual Segmentation	Alienware Aurora R8 (Dell) with an Intel Core i7-9700 processor, a NVIDIA GeForce RTX-2080Ti, and 16 GB of RAM	MRI	VR	5 medical professionals ((4 physicians: 1 fourth-year resident, 1 third-year cardiac fellow, 1 pediatric intensivist, and 1 pediatric cardiac surgeon) and 1 biomedical engineer)	1	time used for desiging bifurcated and tube-shaped grafts, evaluation of the design	none	yes
Virtual reality digital surgical planning for jaw reconstruction: a usability study	Manzie et al.	2023	usability study	otorhinolaryngology/oral and maxillofacial surgery	ablation and reconstruction of head and neck pathologies	custom made	Oculus Quest 1	Meshes	Yes	Alienware A51m x64-based laptop (Intel(R) Core(TM) i7-9700 CPU @ 3.00Ghz, 8 Core(s), 64 GB RAM, and NVIDIA(R) GeForce RTX 2080)	not reported	VR	13 (9 oral and maxillofacial surgeons, 2 otolaryngology surgeons, 2 general surgeons trained prior to post-fellowship training in head and neck surgery)	1	usability	none	yes
Evaluating Soft Organ-Shaped Tangibles for Medical Virtual Reality	Muender et al.	2023	usability study	general surgery	liver surgery	custom made	Valve Index	Meshes	Manual Segmentation	Intel i7 processor and a Nvidia 2080 TI graphics card	MRI	VR + 3D printed model	13 (assistant doctors (n = 5), consulting physicians (n = 2), senior/head physicians (n = 6))	2	user experience, work load, interaction	none	not applicable
Evaluation of voice commands for mode change in virtual reality implant planning procedure	Rantamaa et al.	2023	usability study	oral and maxillofacial surgery	implant planning	custom made	Oculus Quest 2	Volumetric	No	no info	CT	VR	6 dentomaxillofacial radiologists	1	objective (completion time, accuracy of position), subjective ratings	none	not applicable

**Table S2**	Sorted by year of publication																
**Title**	**Authors**	**Year of publication**	**Study design**	**Surgical discipline**	**Procedure / indication**	**Name of VR-software**	**Head-mounted display**	**Rendering technique**	**Segmentation**	**Computer**	**Imaging input for VR model**	**Visualization modalities, comparisons (versus) and presentation order (then)**	**Participants**	**No. of cases**	**Outcome measures**	**Comparison of VR with...**	**Favours VR**
Evaluation of virtual handles for dental implant manipulation in virtual reality implant planning procedure	Rantamaa et al.	2023	usability study	oral and maxillofacial surgery	implant planning	custom made	Oculus Quest 2	Volumetric	No	no info	CT	VR	4 dentomaxillofacial radiologists	3	usability	none	not applicable
Fully Automated Conversion Of Glioma Clinical MRI Scans Into A 3D Virtual Reality Model For Presurgical Planning	Tucker et al.	2023	software elaboration + user study	neurosurgery	glioma surgery	Enduvo	not reported	Volumetric	AI/Automated Segmentation	Intel Xeon CPU E5-2620 v2, 2.10GHz, 64 GB RAM, and GPU NVIDIA GeForce GTX 1060 6GB	MRI	VR	2 neurosurgeons	model was trained on the Brain Tumor Segmentation (BraTS) challenge data set (Menze et al. 2015, Bakas et al. 2017)	required time, AUC	none	not applicable
